# Paving the Path Toward Retirement for Assistance Animals: Transitioning Lives

**DOI:** 10.3389/fvets.2019.00039

**Published:** 2019-02-21

**Authors:** Zenithson Ng, Aubrey Fine

**Affiliations:** ^1^Department of Small Animal Clinical Sciences, University of Tennessee College of Veterinary Medicine, Knoxville, TN, United States; ^2^Department of Education, California Polytechnic State University, Pomona, CA, United States

**Keywords:** retirement, aging, assistance animals, service animals, welfare

## Abstract

Assistance animals play significant roles in human therapy and well-being and represent a rapidly growing demographic of animals in society. Most research in the field of assistance animals has been focused on the effect of these animals on people. Only recently has there been a growing interest in the welfare and well-being of these animals and the effect of the work on the animals themselves. The concept of retirement, or withdrawing the animal from its working life, is an important welfare consideration that has received minimal discussion in the scientific literature. The notion of retirement is typically regarded as a reward earned after a lifetime of work, but this inevitable phase of an animal's working life has positive and negative implications for both animal and handler. Some of these implications include recognizing the emotional impact of this life-altering event on both animal and handler. The decisions of when and how to appropriately retire an animal are typically made at the discretion of the assistance animal agencies and handlers, but standard evidence-based guidelines for the proper retirement of assistance animals are currently unavailable. This review will provide considerations and recommendations for the retirement that assistance animals deserve.

## Introduction

*Recently, I watched an older mobility service dog join her family on the plane. Her handler had some significant mobility impairments, and it was clear that her dog was a tremendous asset to her. As they got settled, we began to talk. She let me know that “Trixie” was getting quite old, and they were planning to retire her in the next 4 to 6 months. They felt that her role as service animal was becoming too demanding for Trixie, and they felt she deserved a break. The woman shared how concerned she was about Trixie's retirement primarily because she wondered how she would handle this new change in life. She noted, “Humans make the choice to retire, and for some of us, we are ready for our new future…” On the other hand, “Our animals don't consent to this process, and I wonder how some will do, including my dear Trixie.” Although Trixie would be retired, she planned to keep her as her family dog, while they would be adopting a new service dog to take on that role. There was never a thought in her mind that Trixie would leave the family*.

The emotional conundrum of retiring an assistance animal is an issue every handler must confront at some point of the animal's life. The cessation of a working career should be perceived as a well-deserved, positive celebration and a guarantee that the animal will enjoy the remainder of his or her life. However, retirement also marks finality and can be a difficult road for both animal and handler to navigate physically and emotionally.

There has been growing attention to the welfare of assistance animals during their active working lives. Despite the fact that every assistance animal will inevitably face the reality of retirement and end of life, research has rarely addressed these issues. Evidence-based guidelines of when and how to appropriately retire an assistance animal are necessary for the welfare of the animal and handler. The purpose of this review is to define the retirement of an assistance animal, describe the implications of assistance animal retirement for both handler and animal, and discuss the challenges in determining when to retire an assistance animal. While the concept of retirement may be applied to any species designated as assistance animals, this review will be specific to the canine species.

## Defining Retirement

Retirement represents one of the greatest lifestyle changes an individual can experience in life. It signifies the beginning of a new era and, more specifically, the beginning of the last phase of life. This topic is of utmost importance in today's world because of technological advancements in medicine permitting humans (and animals) to live healthier and longer lives than ever before (1). Of course, longer lives lead to longer retirement (1). This period represents a significant portion of the animal's life that should receive special attention because of its aging physical state. While retirement is exclusively a human concept, it certainly applies to the life of working animals.

Retirement is the ultimate respite from the work an assistance animal performs. An assistance animal may spend its retirement in the home of the handler he or she has been assisting, in the home of the individual who raised the dog as a puppy, or in the home of another individual screened and approved by the agency from a waiting list (2). However, retirement is spent, the animal should be free of obligations and be simply considered a pet that belongs to an owner, rather than a medical device specifically trained to perform a task for an individual with a disability.

This, however, does not mean that the animal is duty-bound to a sedentary, isolated lifestyle. The animal should continue to remain active, stimulated, and engaged in a manner that is not distressing, with adequate environmental enrichment (3). Instead of full retirement, the animal may also enter semi-retirement, in which the animal retires from his or her full time assistance animal role but still works part time at a reduced capacity. This is likely not an option for the handler that requires an assistance animal full time. Rather, the animal can transition to a different, less demanding career in semi-retirement as a therapy dog, search and rescue dog, or detection dog. The frequency, duration, and intensity of work can gradually decrease to complete cessation of work and full retirement, depending on the response of the animal. Some working animals are rejuvenated with a new role. For example, a military working dog diagnosed with post-traumatic stress disorder (PTSD) after deployment was not fully retired, but rather trained to be a service dog; this shift and new sense of purpose reportedly eased this dog's PTSD signs (4). However, retirement is lived, it should be a positive experience, ensuring good quality of life until the animal's end of life.

## Implications of Retirement

Retirement of assistance animals has both positive and negative impacts on the well-being of both the animal and handler. A great deal of research has been conducted concerning the psychosocial and health ramifications of retirement on humans, but little research has specifically addressed issues regarding the retirement of assistance animals. The term “retirement adjustment” is used to describe when people prepare and become accustomed to the changes associated with the transition from work to retirement (5) and can be applied to assistance animals as well. With proper planning and maintaining a healthy lifestyle, retirement should be a positive experience.

### For the Animal

In retirement, the animal no longer has obligations or duties to fulfill; life is more calm, less stimulating, and perhaps less stressful for the animal. Factors, such as unintentional maltreatment of the animal, overstimulation from humans other than the handler, lack of predictability in daily routines, and insufficient opportunities for recreational activities, have been reported to be welfare concerns for service dogs (6, 7). Because of the nature of their work, these dogs may also be exposed to adverse environmental conditions and the transmission of zoonotic and other infectious diseases (8). Retirement provides freedom from these stressors of working life.

While it may be perceived that retirement is a liberation of sorts, this human sentiment of retirement may not be similar to how assistance animals experience retirement. Assistance animals may not necessarily perceive their working roles as arduous work they wish to escape in the traditional sense that some people do. Retirement can, in fact, be distressing for some animal retirees because the transition from working full time to not working at all can be a dramatic and challenging adjustment.

Potential negative implications of retirement for the animal may be extrapolated from the human literature. Various studies in humans have shown that retirement resulted in negative effects on physical health (9, 10) and declines in mental health characterized by decreased well-being and increased depression (11–13). Being forced into retirement while still having a strong desire to continue working or feeling without a purpose are negative outcomes that some may struggle with during post-work life (14). People who have worked for decades may not realize they are no longer physically or mentally capable of the same type of work that was part of their daily routine for most of their adult years. Assistance animals have been trained to perform certain tasks and have been performing them for most of their lives. In essence, it becomes part of their nature, and their work habits are embedded into their daily lives, potentially making them harder to break because they do not see a life without them. Retirement may be perceived as a disengagement from their typical life routines because they do not know a life that is any different. Such a dramatic change may not only be confusing, but also emotionally taxing.

How the animal responds to this life change likely depends on the drive of the individual animal. Houlfort et al. (15) points out that the type of relationship a person had toward work, whether “harmonious or obsessive,” will be a factor in his/her mental adjustment to retirement. For example, a person who has an obsessive need to work will not adapt as easily to retirement as someone who has a less intense need for work satisfaction (15). Every individual is different, so while one obsessive assistance dog with high drive may have a difficult time adjusting to retirement, another slothful assistance dog with lower drive may adapt to retirement without issue. However, the majority of assistance dogs are selected because of their “drive” to work and thrive when they have a specific purpose and role (16). The lack of purpose can be distressing to these dogs that crave constant attention and purpose. The dog may continue to perform tasks he or she is accustomed to doing, such as picking up objects when they are dropped (as a previous disability assistance dog) or barking when the phone rings (for a hearing dog). When the task is not rewarded or perhaps discouraged in retirement, the dog may become frustrated and anxious, leading to maladaptive behaviors indicative of poor welfare. In essence, the dog may need to be re-trained to not function as a working dog. As we begin to understand more about canine cognition, we may discover that the loss of purpose associated with retirement has an emotional toll on dogs (17, 18).

The difference between retirement for people and retirement for working animals is that people can consciously anticipate it and understand why they are no longer working. Animals, however, live moment to moment and are unable to be comprehend that they will retire in the future and do not understand the reason why they are no longer required to work. While people have control of their own lives and have the will to decide when and how retirement will commence, animals are unaware this is even an option since this fate is determined by the handler and/or assistance animal agency.

Where the animal spends its retirement also depends on the handler and/or assistance animal agency. Specific changes to daily routine in retirement largely depends on whether the animal retires in the home of the handler or in a different home without the handler. When the animal retires with the handler, the challenge for the animal to adapt to is no longer accompanying the handler at all times in all locations. The animal is likely strongly attached to the handler, and the separation can be stressful, especially when the animal does not understand why this is happening. Signs of separation anxiety may manifest because the animal is now left home alone without the social interaction or attention he or she has been accustomed to for the majority of their life. In addition, since the assistance dog accompanied a human at all times, the needs of this dog were attended to constantly, such as the need to be walked, fed, watered, and played with. When the dog is left home alone in retirement, there is the potential for the dog to receive less diligent care and attention. It is essential that provisions and services, such as regular dog walking or alternative means of care, be provided for the retiree. One of the most significant stressors for the animal retiring in the home of the handler can be the introduction of the new animal that will be replacing him or her. Particularly in a single dog household, the addition of a new dog may present problems resulting in inter-dog aggression and other maladaptive behaviors.

The stress of coping with retirement may be mitigated or magnified if the animal is taken out of the home and assigned to live with a new owner. A dog may not be as confused if the handler he or she has been working with for their whole life is no longer present. If the retirement home is the original puppy raiser or another person the dog is familiar with, the dog may be able to adjust more comfortably. Conversely, a dog may not adjust to a new home very well, not only because of the change in routine, but because of potential changes in numbers and types of people in the household, numbers and types of other animals in the household, and differing home environments (i.e., climate, flooring, physical space, etc.).

Since assistance animals typically do not interact with strangers while working to avoid distraction, they have not been accustomed to unfamiliar humans interacting with them. In retirement, these dogs will likely be approached and pet by strangers, which may be confusing to the dog. Studies have shown that older dogs cope less efficiently to stress caused by mild social challenges (19). Aged dogs in this study behaved more passively, showed less interest in interaction with a stranger, and demonstrated a significantly increased physiologic stress response after exposure to a stranger. This indicates that older dogs may not be as adept at managing social situations. This may be particularly true for older assistance dogs that have retired, which can certainly impact their emotional well-being. For this reason, the process of entering retirement should be carefully planned and modified according to the response of that particular animal.

### For the Handler

It is no surprise that the retirement of an assistance animal affects the handler just as much as, if not more than it affects the animal itself. The period of transition into retirement may cause some handlers an enormous amount of stress, difficulty, and pain because of the strongly established bond (20). Wrobel and Dye (21) also suggest that the bond between the assistance dog and handler is unequivocally strong, and the process of grieving due to retirement or death of the animal may be significant. The ramifications may impact mental health and activities of daily life.

There is a plethora of research that highlights the physiological and psychological significance that animals provide to humans (22, 23). It is only logical to assume that the benefits found in humans' relationships with their companion animals would be similar or even more substantial in the relationships with individuals requiring assistance animals. Sachs-Ericsson et al. (24) highlighted numerous studies demonstrating that the assistance animal provides for not only the individual's enhanced independence, but in promoting the individual's psychological well-being as well. Camp (25) reported that numerous individuals who have assistance animals identify their relationship with the dogs as one of the most important benefits of the relationship, oftentimes superseding the functional tasks the animals perform. Lane et al. (26) reported that most owners consider their assistance dogs a critical member of their family, instead of just a working dog. The emotional significance that these animals have in the lives of the humans they support is crucial in understanding why individuals may experience tremendous hardships when retiring their assistance animals. In essence, the handler feels very connected to his animal. He or she has learned to rely on that animal over the years to live his or her life.

In addition, attachment theory provides additional insight into the reason why humans are so attached to companion animals and particularly the assistance animals they care for. Attachment theory was developed by John Bowlby (27), who described the major element in parent-child relationships as attributed to humans' desire to protect their infants. This theory suggests that our strong relationships with animals exist because of our innate attachment needs as caregivers. Zilcha-Mano et al. (28–30) suggest that humans view their companion animals in a similar fashion as those taking care of an infant. Furthermore, Kwong (31) discovered that caregiving was an important dynamic in the development and preservation of the relationship between assistance dogs and their human counterparts. Recipients of assistance animals are taught early in their training with their selected animals that caring for and engaging with the animals is essential for a strong human-animal bond that forges an effective working alliance. This helps to understand why a handler can have a difficult time coping once the working alliance is terminated upon retirement of the assistance animal. Folk (32) believes that it may be harder on the human partner than the dog because it represents a significant adjustment in the human's everyday life.

Once the decision to retire an assistance animal is made, the impact of this change on the handler will depend on whether the animal spends retirement with the handler or with a new family. While a handler may desire to keep the retired animal while integrating a new assistance animal, there may be numerous challenges to the transition. The indication for the retirement of the animal may be due to the development of a physical illness that requires advanced care. This care may include frequent veterinary visits, medication administration, and implementing special accommodations that the assistance animal may need. The handler may be unwilling or inept to attend to animal's needs (i.e., life changes or physical handicaps the handler may be experiencing that interfere with the ability to provide for the animal). Additionally, it is emotionally draining for a handler to watch a previously robust assistance animal age, slowly decline in health, and inevitably make end of life decisions on behalf of the beloved animal.

For these reasons, many handlers are comfortable with retiring their assistance animal to a good home. However, Folk (32) believes that the handler may begin to feel a sense of guilt about relinquishing the assistance animal. She explains that an individual may feel that relinquishment may be similar to abandoning their companion at the end of his or her life after all that the dog has done for him or her. The sentiment that the dog will ultimately be adopted into a dependable home that is guaranteed to meet all his or her needs to ensure a healthy and fulfilling retirement may comfort these handlers during the separation. While some may find consolation in visiting the assistance dog in his or her new home during retirement, others may find it difficult to only visit for a short period of time. Although the handler should logically perceive that retirement is unavoidable and in the best interest of the animal, the handler copes with many logical hardships during the transition.

## When to Retire an Assistance Animal

An animal should retire when, and preferably before, it exhibits physical or mental health conditions that impair its ability to work. Currently, there are no evidence based studies nor standard, established guidelines that indicate when an assistance animal should retire. Assistance Dogs International (ADI) is a leading international umbrella organization that provides guidance and membership for approved non-profit programs that train and place assistance dogs. As a leading authority, assistance dog agencies seek their counsel on assistance dog issues worldwide. ADI standards do not state specifics regarding the retirement of assistance dogs (33). According to Gorbing (34), secretary of ADI, “There are no mandatory standards around the retirement of assistance dogs within ADI, although to some extent, the issue is addressed through some of the other standards e.g., the need for annual follow-up by programs on all of their clients, including the requirement to obtain a veterinary report assessing the dog's fitness to remain working.” Regardless, universal guidelines are non-existent for those seeking formal recommendations.

In situations where the dog is still owned by the assistance dog agency, each agency may have their own parameters to determine when the animal is ready to retire, typically based on veterinarian reports, annual reports from handlers, and site visits (2). However, there is no oversight for the retirement of dogs of agencies that completely transfer ownership to the handler or dogs that are individually trained and owned by their handlers. Gorbing (34) also notes that “programs also have a mandatory duty to prepare clients for the retirement of the dog at some stage through the provision of information and support. In practice, it is always a tricky issue to deal with, but if programs start from an understanding of what is best for the dog, from my experience, it is fairly clear when the point of retirement comes.”

### By Health Status

The clearest indication for retirement is the presence of any disease that inhibits the animal's ability to work. Any change in the animal's physical health warrants veterinary evaluation and cessation of work until the problem is addressed and resolved completely. One study investigated the incidence of health conditions, time of retirement, and cause of retirement in 7,686 guide dogs in the UK (35). The most common causes for early retirement in guide dogs were musculoskeletal and neurologic conditions (35). Clinical signs of musculoskeletal disease include slowing down, weakness, difficulty getting up and down stairs, and challenges with rising and lying down. Osteoarthritis was the main cause and diagnosis of musculoskeletal signs that mandated retirement (35). Clinical signs of neurologic disease include seizures, circling, falling, and paraparesis. Epilepsy was the main cause and diagnosis of neurologic signs that mandated retirement (35).

The assistance animal may succumb to a multitude of other conditions affecting body systems that impair his or her ability to work. Clinical signs of cardiorespiratory disease include excessive coughing, increased respiratory rate, difficulty breathing, weakness, and collapse. These signs warrant immediate attention as they can be indicative of rapidly fatal disease. Clinical signs of gastrointestinal disease include vomiting, diarrhea, decreased appetite, and weight loss. Although many gastrointestinal conditions may be temporary and likely due to dietary indiscretion, signs that are chronic in nature warrant retirement. Clinical signs of urinary disease include urinary incontinence, increased drinking, increased urination, and straining to urinate. Because these signs may be linked to endocrine diseases, thorough veterinary investigation is necessary. Clinical signs of dermatologic disease include scratching, rashes, and skin masses. Interestingly, dermatologic conditions caused by atopic dermatitis were the ailments that reduced the working life of guide dogs the most, by an average of 5 years (35).

Impairments of the senses, specifically vision and hearing, critically affect the life of an assistance animal. Clinical signs of ophthalmic disease include difficulty navigating, sudden blindness, clouding of the eye, excessive ocular discharge, and redness of the eye. Clinical signs of hearing loss include decreased reactions to sounds or verbal commands, which can be more difficult to appreciate. In general, any physical changes in the animal's health including weakness, lethargy, change in activity or rest, and changes in performance should be addressed immediately. Most importantly, any conditions that cause significant pain to the animal warrant cessation of work. The animal should not be forced to work while trying to recover from a health condition and veterinary guidance is necessary to determine if and when the animal should return to work.

Changes in behavior warrant a veterinary evaluation and consultation with a behaviorist. Clinical signs including aggression, vocalization, atypical behavior, changes in attitude, and disorientation may indicate a behavioral disorder, but systemic medical conditions must be ruled out first. Behavioral changes may actually be due to neurologic, endocrine, or pain-related conditions. Furthermore, older dogs that exhibit changes in mental status may suffer from the canine equivalent of Alzheimer's disease, which is called canine cognitive dysfunction. Cognitive dysfunction is prevalent among 14.2–22.5% of all geriatric dogs (36, 37). The condition is characterized by altered sleep cycles, decreased social interactions, disorientation, anxiety, and house soiling. Although the disease progression can be delayed and managed, the condition is irreversible and clearly impacts the assistance dog's ability to work, making retirement necessary.

In addition, handlers and veterinarians should monitor for behavioral signs of stress and anxiety. Signs of stress commonly exhibited in dogs include increased restlessness, snout licking, paw lifting, yawning, body shaking, nosing, circling, increased locomotor activity, and lowering of body posture (38, 39). An increase in these subtle behavioral indicators of stress while working may be the first sign that retirement should be considered. Therefore, handlers must pay careful attention to any trends in these signs.

### By Age

While these changes in physical health and behavior indicate consideration for retirement, the ideal retirement should be mandated long before signs of illness ensue. This presents a challenge because an individual may not feel that a healthy animal needs to be retired, especially when it is fully functional at its work. However, the animal should retire in order for it to enjoy retirement in good health, rather than in a debilitated state. The ideal duration of time the retired animal should be in good health is unknown, but it is reasonable to consider when the animal reaches the senior life stage, defined as the last 25% of the dog's expected lifespan (40).

To address the challenge of retiring a healthy animal, some individuals may be inclined to use age as the major factor for retirement. However, using an age cut-off may be unreliable given the varying life expectancies of species and breeds. For example, it is widely accepted that larger breeds of dogs have shorter lifespans than smaller breeds (41, 42). Of course, this is not always the case since genetics and preventive health care practices play large roles in lifespan. For example, a chihuahua expected to live to 16 years could be cut off to retire at 12 years of age to achieve 25% of life in retirement but could pass away at 13 years of age due to an unforeseen condition. Alternatively, a great dane expected to live to 8 years of age could be instructed to retire at 6 years of age to achieve 25% of healthy life in retirement but could live to 12 years of age. Therefore, these would have been inaccurate choices to make.

Age is just a number, and this crude assessment of age as a determinant of retirement assumes that the animal is experiencing healthy aging. Healthy aging is a normal process of life that can be defined as cognitive and behavioral health in conjunction with normal function of individual body systems (43). Even in the absence of disease, normal age-related changes, such as graying of the muzzle and moderate reduction in activity, are bound to occur (44). Healthy aging is also associated with behavioral changes, such as a decline in attentiveness (45), play level, and response to commands (46). Interestingly, healthy aging dogs also change from spending a lot of time interacting with humans to simply spending more time near humans (47). Therefore, a handler should not be surprised that an older assistance dog may choose simply to be around, but not necessarily engage with the handler. This healthy aging phenomenon should be distinguished from senescence, which is defined as the collective, deteriorative changes that negatively affect an aged dog's quality of life (44). Signs of senescence may include osteoarthritis and impairments in vision, smell, and hearing (48). Regular veterinary consultation is essential for every assistance animal to determine healthy aging. Ideally, every assistance animal should be assessed biannually, especially as the animal approaches the senior life stage.

Most service and working dogs, which are typically Labrador Retrievers, German Shepherds, and Golden Retrievers, are estimated to have an average working life of 8 years (35, 49). Since most working dogs do not officially begin their careers until 2 years of age, they are typically retired at around 10 years of age. Because these breeds of dogs have a typical life expectancy of 12–14 years, retirement at 10 years is consistent with the understanding that an animal should retire when it reaches 3/4 of its lifespan. Another study demonstrated that factors associated with early death in guide dogs were an elevated alanine aminotransferase (ALT, a liver-associated enzyme measured on routine bloodwork) and evidence of skin nodules (50). Therefore, veterinarians should routinely assess bloodwork and closely examine the skin in assistance dogs to properly assess their health statuses. Perhaps an assistance dog should be retired earlier than expected if the dog has evidence of elevated ALT or skin nodules since there is a possibility the dog will have a shorter life than the average asssistance dog.

### By Alternative Assessment Tools

Determining when an assistance animal should retire is unclear, multifactorial, and dependent on the individual. Therefore, this decision should be based on careful assessment by the handler/owner in conjunction with veterinary and behavior consultation. To make an informed decision, the veterinarian or behaviorist should be familiar with the duties, working conditions, and potential stressors of that particular assistance animal. However, access to additional parameters that assess animal welfare may help in making a better informed decision regarding retirement.

Objective diagnostic tools to determine appropriate time of retirement would be helpful but do not currently exist. One potential parameter that could be measured is cortisol level, representing the dog's level of stress. While measuring cortisol in saliva, blood, or urine samples reflect acute stressors, these methods may not be helpful when assessing the animal's overall welfare and well-being, since it only represents one point in time (51). To overcome this challenge, cortisol can also be measured in hair samples and reflects chronic stress levels because cortisol accumulates in hair over time (52, 53). This is a relatively new area of study, and numerous factors impact hair cortisol levels. Therefore, universal cutoff levels signifying high stress for assistance dogs cannot be recommended at this time. However, hair cortisol may be measured biannually or annually to assess trends within the individual. If cortisol levels increase significantly from baseline levels, further investigation for underlying disease or chronic stress should be pursued. If the elevated hair cortisol level is associated with behavioral changes in the work, there should be recommendations for either retirement, temporary break from work, or change in lifestyle.

The handler is the most important advocate of the animal's welfare since he or she knows the animal best. The decision to retire an animal, however, is currently quite subjective and biased not only because of the personal relationship with the animal, but also because the handler is strongly bonded to the dog. Handlers have the potential to have their own emotional attachment to the animal assistance work or perceive assistance animals simply as “medical devices,” rather than living entities with welfare needs. Therefore, the handler should remain as objective as possible when assessing the assistance animal for retirement from work. A survey on assistance dog quality of life (QoL) may be a useful tool that can assist the handler in considering retirement in an aging animal that is free of clinical disease or pain. Many QoL surveys and scales have been developed to assist pet owners in deciding when to euthanize an animal, but no scale has yet been established for healthy retirement. Because QoL scales are subjective and not correlated with objective clinical outcomes, their validity and reliability should be interpreted cautiously (54, 55). The proposed scale for assistance dog QoL (Appendix 1) should only be used when the animal is free of clinical disease or pain. Any animal with disease or pain should be automatically relieved of work duties. The survey requires the handler to objectively assess 10 factors that characterize QoL. The survey should be taken while the animal is in optimal working capacity to provide a baseline score and then retaken when retirement is in question. Since QoL is very specific to the individual, rather than using a standard cutoff value, a decrease of 25% or greater from baseline warrants the consideration of retirement in conjunction with veterinary and behavioral consultation, as this instrument is intended to detect subtle declines in QoL. Therefore, this instrument is not intended not be used in isolation, but rather as an impetus to begin a dialogue about the dog's working life with a veterinarian, behaviorist, or other animal expert (56).

## Retirement and Beyond

There are many factors to consider to ensure that the animal is properly taken care of during his or her older years. The designated caretaker of the animal during retirement maintains a critical role in the animal's health and welfare. According to *Guiding Eyes for the Blind* (57), the handler has the opportunity to adopt his or her retired assistance animal or place the dog with an approved adopter. The adopter could be a close friend or family member, which could facilitate a continued relationship with visits (58). If this option is not possible, the retired service animal has the opportunity to return to his or her original foster family who reared the dog during the early training years. In the event that none of these options is possible, the assistance animal may be put up for adoption. Typically, senior assistance dogs are very desirable because of their expert training and calm temperaments. Consequently, there is typically a very long waiting list to adopt a retired dog. Additionally, adopters may be held accountable to follow strict criteria that are put in place by the agency before adopting the dog. For example, some agencies indicate that if you adopt a retired assistance dog, the dog is not allowed to be home alone for more than 4 hours at a time. This oversight helps to ensure the responsibility of the caretakers and thus quality of life for the animal.

As the handler prepares for the animal's retirement, he or she will likely obtain a new assistance animal. One should consider the impact of introducing a new dog into the family on the retiree. Some may expect that the new assistance dog will learn behaviors from the retiring dog, but this cannot be guaranteed. The handler should have realistic goals for the new animal and not expect the new animal to function like the previous one. In addition, while some dogs may enjoy the company of a new conspecific, an aging dog may not be as accepting of a young dog's presence in the home. The aging dog will likely be less active than the new dog and may encounter interdog aggression (59) or even jealousy, especially if the retiring dog perceives the handler giving more attention or rewards to the new dog (60). These adverse events can negatively impact the animal's welfare in retirement. Managing the relationship between household dogs through proper introductions and diligent observation and intervention is essential.

The assistance dog that is retired at the appropriate time will experience a significant portion of time in good health in his or her senior years. Unfortunately, mortality is inevitable and will most frequently be due to neoplasia, musculoskeletal, and neurologic conditions (61), with neoplasia being the most common cause of death in large-breed dogs (62). The loss of any animal is difficult for an owner, but the loss of an assistance animal is particularly challenging because of the nature of the attachment, the role the animal had in assisting the handler in functioning, and the strong bond between handler and animal (63, 64). The owner should be prepared for end-of-life decision making by defining specific criteria for end of life and objectively assessing the animal's QoL in consultation with a veterinarian. This QoL assessment for end-of-life (65) is different from the QoL assessment for retirement. The decision is never easy and requires the selfless regard for animal welfare. Since these animals have devoted a lifetime of work to a human being, the most difficult, yet most noble, decision we can make for them is to elect humane euthanasia before unnecessary pain and suffering occur.

Overall, these animals should be highly regarded and treated with the utmost respect in retirement. They should be spoiled, loved, and permitted to do whatever they please as a reward for the lifetime of service they have given. It could be argued that assistance animals should be honored the same way that military veterans are honored. Like a military veteran who has devoted his or her life to his or her country, an assistance animal has devoted his or her life to a human in need. These retired heroes should be clearly identified with gear such as bandanas or collars that state “retired assistance dog” to indicate to the public that this animal deserves special attention. These animals should receive the benefits of exceptional healthcare in retirement, especially if any conditions were the result of the service they provided.

## Areas for Future Study

Although retirement is a phase of life granted to every service and working animal, research has rarely investigated questions regarding this particular issue. The authors believe the reason for this is 2-fold. First, the need for this type of research has not been imperative because retirement is a process that most handlers and agencies accept, facilitate, and value. Many assistance animal agencies have been managing their programs and endorsing retirement for generations with few reports of adverse events or concerns for animal welfare. In addition, the limited funding available for assistance animal research is typically allocated toward studying the benefits to the human recipient or the factors that produce a successful assistance animal. When an assistance animal retires and becomes a pet, that animal realistically loses its inherent value and purpose to an agency. With this in mind, efforts have not been thoroughly justified to study animals in retirement. As the field pays more attention to the health and welfare of working animals and advances in science and knowledge permit more insight into cognition and biomarkers of stress, researchers and funding agencies may be compelled to conduct and support this type of research to further enhance our scientific knowledge and understanding of the lives of these animals. Secondly, quality research on these retirement issues is challenging to execute because it is a complicated area of study. The questions regarding the specific timing of retirement and impact of retirement on the dog and handler are multifactorial and dependent on the individual animal, handler, lifestyle, work, genetics, and countless other variables that are difficult to control. The inability to control for real life factors limits the conclusions that can be drawn. Despite this complexity, research is still justified, even with its transparent weaknesses.

The main priority for research in this topic should be determining the appropriate timing of retirement of assistance dogs that best maximizes the dog's working life while still guarantees a healthy retirement. Currently, the timing of retirement is relatively subjective. Objective measures such as hair cortisol and instruments such as the QoL discussed previously may be used in research to assess their utility, accuracy, and validity in retirement determination. In addition, there is a lack of understanding of the lifestyle of retirement that ensures the best quality of life for that animal. Whether the dog should be fully or semi-retired, whether the dog should retire with the handler or original puppy raiser or unfamiliar home, and whether the animal should retire with other animals in the household are just some of the questions that exist. Investigations may reveal how these factors influence or are associated with the animal's QoL, emotional health, physical health, and longevity. These findings will provide evidence-based guidelines of when and how to retire an assistance animal to ensure the best welfare and quality of life for that animal. Agencies without standard guidelines for retirement may refer to these; agencies with already established guidelines for retirement may modify their own to align with evidence based guidelines. However, each agency must consider the unique needs of their particular demographic of handlers, breed of dogs, type of training, and type of work when implementing universal guidelines. Some recommendations may be appropriate while others may not be, so animal welfare experts familiar with the specific details of that agency should critically assess and modify standard guidelines to best meet their needs.

On the human side, studies should explore the handler's experience when preparing for and adjusting to an assistance animal's retirement. The transition can be challenging for the handler, but positive emotions, such as a feeling of relief that the animal is free of responsibility, may be evoked. Through interviews and surveys of handlers, an understanding of their challenges and successes can inform agencies how to properly counsel their clients on what to expect and how to cope with an animal in retirement.

While some research has explored the retirement of guide dogs by established agencies, to the authors' knowledge, there is no information regarding the retirement of assistance dogs that are individually and independently trained and owned. Any individual with a disability may be permitted to train and designate any type of dog as an assistance animal. This freedom to label an owner-trained dog as an assistance dog along with the challenges of acquiring an agency trained assistance dog have resulted in the rapid growth of self-proclaimed service animals and enable a wide variety of problems. Without oversight or guidance by experienced agencies or organizations such as ADI, the welfare of these animals may be at risk and retirement may not even be considered. Without standard guidelines, these independent handlers have little incentive or reason to retire their dogs (34). An investigation into the attitudes and practices of these handlers is difficult to achieve because they do not belong to a universal community that can be easily contacted. If investigators were able to capture this specific population of self-trained animal handlers, we would be able to understand the concerns that surround retirement among this unique category of animals.

The process of retirement is fraught with unique rewards and challenges humans and animals alike. Controlled retrospective and prospective studies should explore this process with the ultimate goal of enhancing the health and welfare for all assistance animals.

## Conclusions

Every assistance animal deserves to be rewarded with a good quality of life both during and after his or her career has ended. The connotation of retirement is inherently positive and enhances welfare because the animal is free to enjoy life without responsibility. However, as discussed, this permanent end of work constitutes a complex emotional process for all parties involved. Both animal and human must confront the dramatic lifestyle changes associated with adapting to a new life without one another or in the capacity as a pet. As the field of human-animal interactions becomes more attentive to the physical and emotional needs of the assistance animal, research to support evidence based guidelines on the complete care of the animal becomes more necessary. Studies that investigate timing of, reason for, and lifestyle of retirement will provide further insight into these critical issues. Even if guidelines are established, however, adherence to them may be challenging. This may be especially true for handlers of those assistance animals that are individually and independently trained by the handler rather than a professional assistance animal agency. These individuals may be unaware that guidelines exist or ill-equipped to properly implement practice standards. Recommendations for retirement may arguably be more important for these independent handlers to adhere to than for agencies that may have their own retirement standards. It is important for these independent handlers to have a common resource for guidance and support. The authors endorse the need for more oversight of all assistance animals, regardless of their origins, to ensure that standard guidelines are sanctioned and the welfare of the animals is prioritized.

The life and welfare of an assistance animal should always be kept in the highest regard. The assistance animal lives a humble life of purpose and can be the essential lifeline for a human being, a fate that nature could have never commanded. There is no question that the assistance animal must be honored treated with the utmost respect in every stage of life. As the animal transitions into retirement, the lives of both animal and handler change, but the memories and history between the two lives do not. Robert Frost (66) once stated, “The afternoon knows what the morning never suspected.” By supporting these hard working beings, we will be able to provide more formidable opportunities for assistance animals to have a better quality of life.

## Author Contributions

All authors listed have made a substantial, direct and intellectual contribution to the work, and approved it for publication.

### Conflict of Interest Statement

The authors declare that the research was conducted in the absence of any commercial or financial relationships that could be construed as a potential conflict of interest.
