# Supplementary material for: Paving the Path Toward Retirement for Assistance Animals: Transitioning Lives
Source: Front Vet Sci. 2019 Feb 21;6:39. doi: 10.3389/fvets.2019.00039 (PMC6393662; doi:10.3389/fvets.2019.00039)
Supplement: Supplementary file 1 [file Table_1.docx]

**Supplemental Material**

**Appendix 1: Assistance Animal Quality of Life Scale**

Place a check mark for what is most accurate for your assistance animal at the current moment. The healthy animal should be free of clinical disease or pain.

|  | **Strongly Disagree** | **Disagree** | **Neutral** | **Agree** | **Strongly Agree** |  |
| --- | --- | --- | --- | --- | --- | --- |
| 1. **Sociability**   The animal is engaged, seeks attention and stays engaged with people and/or animals when permitted |  |  |  |  |  |  |
| 1. **Enthusiasm for work**   The animal appears excited to travel, wear any working apparel/equipment, or observe any cues that he/she is going to work |  |  |  |  |  |  |
| 1. **Playfulness**   The animal spontaneously engages in play when permitted |  |  |  |  |  |  |
| 1. **Energy level**   The animal exhibits an appropriate level of energy for the activity he/she is engaging in |  |  |  |  |  |  |
| 1. **Rest**   The animal sleeps with ease and appears alert and well rested when awake |  |  |  |  |  |  |
| 1. **Mobility**   The animal walks, runs, jumps, rises, and lies down with ease |  |  |  |  |  |  |
| 1. **Appetite**   The animal has a regular and consistent appetite |  |  |  |  |  |  |
| 1. **Predictable eliminations**   The animal always urinates and defecates when expected without accidents or incontinence |  |  |  |  |  |  |
| 1. **Obedience**   The animal responds to commands immediately and consistently |  |  |  |  |  |  |
| 1. **Minimal displays of stress signals**   The animal does not exhibit signs of stress (i.e., excessive lip licking, yawning, pacing, crouching, circling, whale eye, paw lifting) during work or rest |  |  |  |  |  |  |
| Total number of checks |  |  |  |  |  |  |
| Multiply by | 0 | 2.5 | 5 | 7.5 | 10 | Total |
| Total | + | + | + | + | = |  |

The survey should be taken while the animal is in optimal working capacity to provide a baseline score and then retaken when retirement is in question. The maximum total score is 100. A decrease of 25% or more from baseline score warrants consideration for cessation of work and retirement.
